# Supplementary material for: Prevalence of refractive errors and risk factors for myopia among schoolchildren of Almaty, Kazakhstan: A cross-sectional study
Source: PLoS One. 2022 Jun 3;17(6):e0269474. doi: 10.1371/journal.pone.0269474 (PMC9165805; doi:10.1371/journal.pone.0269474)
Supplement: Attachment — Submitted filename: Response to reviewers.docx [file pone.0269474.s001.docx]

PONE-D-21-32174

Prevalence of refractive errors and risk factors for myopia in schoolchildren of Almaty, Kazakhstan: a cross-sectional study

PLOS ONE

To the Editor:

We appreciate the reviewers’ responses and the Editor’s comments. In the following detailed response, we address each critique calling for changes point-by-point, indicating where relevant additional text has been added to the body of the manuscript and its location. We hope that this has improved the manuscript.

Reviewer #1:

1. Questions:
   Line 43-45: mix data from different age groups to reach the conclusion of "Southeast Asia has been shown to have the greatest fraction of population with myopia and astigmatism whereas hyperopia in children ..." can be misleading, especially when this sentence is following the statement of Line 41-43. Please focus on the results of children studies.

*We thank you for your comment and agree that the sentence was misleading. Therefore, we have rephrased the sentence to read: ‘**Childhood myopia takes the lead in some countries of Southeast Asia with prevalence reaching 80% among adolescents, whereas hyperopia in children may be most prevalent in the Americas’.*

2. L189: Typo, should be “Table 1” here?

*Yes, thank you for catching this up. Now corrected to "Table 1"*

3. L195: this paragraph is the description for Table 2, The prevalence of different types of refractive errors by gender, school types and grades

*Thanks for the comment. Indeed, that paragraph describes table 2, and have now amended the body text to be more consistent with the corresponding Table. Now this paragraph reads: “The overall prevalence of myopia was 28.3% (95% CI 26.5; 30.1). Of all participants with myopia, 79.2% had low myopia, 16.4% had moderate myopia, and 4.5% had high myopia. High myopia was more frequent among students of gymnasiums than among general education schools (6.5% vs 2.7%, p=0.014), while low myopia was more often observed in students at general education schools. Prevalence of hyperopia was 3.4%. Low hyperopia was found in 89.6% and moderate hyperopia was detected in 10.4% of all hyperopic participants. As demonstrated in Table 2, there were no significant differences in the prevalence of myopia and hyperopia among boys and girls.”*

Reviewer #2:

Abstract:

1. introduction: It appears that a study on refractive error prevalence has been published before? <https://articlekz.com/en/article/24144> This statement needs adjusting as it is a rather bold claim.

*Thank you for your important note. This article was also published by us, in which we showed the intermediate results of the research (pilot project), which included a limited number of children and schools. In addition to a greater number of respondents, our current study also examines risk factors for myopia. We also edited the introduction with the aim to better integrate preceding data from Kazakhstan.*

2. Line 25 methods: What is a school shift? This wouldn’t be generally understood.

*In Kazakhstan, as in other countries of the former USSR, public schools have a double-shift system. In a double-shift system, schools divide all covered population into two groups. Each group uses the same buildings, equipment and teachers, but at different times of the day. Pupils of the first shift usually attend school from early morning until mid-day, whereas the second group in in classes from mid-day to late afternoon. However, there also exist schools with full-day classes where children stay in class from morning to evening. In the present study, we compared two shifts with each other, because there is a difference in lighting, daylight hours and daily routine (time to wake up and go to bed). We have now provided more information on that in Methods within the body text, not in the abstract.*

3. Line 33 Results: Large claim that time outdoors and sports are protective against myopia. None of the research in this paper can validate that claim, as it is just a cross-sectional observational study. I appreciate the context within other papers and the theory, but you cannot write this in the results and should be cautious of it again in your conclusion.

*Thank you for your comment. We agree that the wording is not accurate. Changed to «were associated with a lower incidence of myopia»*

4. Introduction:
   Line 41: myopia being the second most common cause of blindness is a worthy point, but given that it can be corrected with spectacles, should this not state that it’s reversible blindness or similar?

*Thanks for the important note! We rephrased this sentence, and now it reads: «Uncorrected refractive errors are the leading cause of moderate to severe visual impairment worldwide and the second most common cause of avoidable blindness»*

5. Line 44: Word choice: fraction may not be best

*Thank you for your comment. The sentence is revised and corrected: «Childhood myopia takes the lead in some countries of Southeast Asia with prevalence reaching 80% among adolescents, whereas hyperopia in children may be most prevalent in the Americas»*

6. Line 49: Although it’s great that you’ve put percentages here to discuss the prevalence in Asia, you did not do this for other comments you’ve made here in the introduction on prevalence of refractive errors such as hyperopia in the Americas. Please remove or add the others percentages for consistency. Furthermore, the studies and prevalence/percentages stated are from studies that include subsets of population, which isn’t representative. For example the 96.5% from Korea is from young men enlisted, and many of these studies do not have rural area representation, which may bias. I’d be cautious here.

*Thank you for your comment. The paragraph has been revised and corrected*

7. Line 51: reword: the greatest burden of refractive error is myopia.

*Thank you for your comment. The sentence has been corrected.*

8. Line 54: The statement of 19.7% of the population being highly myopic in 2050 is wrong from the source claimed. Please revisit and revise.

*Thank you for your comment. We do apologize for the typo. Corrected by 9.8%*

9. Line 60: these are really interesting points on the lack of publication and the differences noted in the former soviet union. Could reasons for this be discussed, is this due to ethnicity, or data collection methods?

*This is indeed a multifactorial problem. It can be assumed that the differences in the data are related both to different geographical and climatic conditions, and to the ethnic and cultural characteristics of the peoples living in the countries of the former USSR. In addition, there are no uniform methods to collect and process data, even within the same country. To elaborate on that, we have now added a sentence in the end of the paragraph which reads: “**Inconsistent data from the countries across the former Soviet Union may results from varying geographical and climatic conditions in addition to differences in ethnic and cultural composition of the peoples living in these countries”.*

10. Line 61: Thus doesn’t fit here as grammatically correct.

*We apologize for the mistake. Corrected to "For example"*

11. Line 66: phrasing such as ‘so far’ is informal. Generally this paragraph is written differently from the others with a colloquial phrasing, and needs changing.

*Thank you for your comment. We have now rephrased the sentence to read: “In the last 20 years, a number of studies reported the prevalence of visual impairments in school-age children in Kazakhstan”*

12. Line 68: these are great prevalence data you’ve discussed, but don’t they show that the statement in the Abstract about this being the first paper to discuss refractive error is wrong!? Also, please expand on these papers, are they from particular age ranges etc? Furthermore, you’ve claimed that the results of these studies differ greatly; reading what you’ve written it’s only 0.6% different. so please check again. I believe you need to rewrite these claims in the introduction as it appears there is data and evidence, but that the general information has key points missing and each study has limitations.

*Thanks for the comment. We have changed the abstract about the primacy of our study. Indeed, studies of visual impairment and refraction among children have been conducted before. Many of them were not published and were used only for internal statistics and reports in clinics. In addition, we have revised and corrected this paragraph to read:* *’A cross-sectional study in the 2004 in Almaty, Kazakhstan revealed a 21% prevalence of refractive errors in schoolchildren, whereas myopia was confirmed in 14%, hyperopia in 3%, astigmatism in 1% and accommodation disorders in 3%.* *Another study (2010) reported that myopic refraction was found in 12% of rural school children and 22% among their urban counterparts.* *Recently, there were only a few studies of the prevalence and structure of refractive errors among schoolchildren in Kazakhstan.’*

Methods

13. Line 84: These comments on the number of inhabitants and ages of children in ages etc. are good but need referencing for their sources.

*We took the data from the website* [*https://stat.gov.kz/official/industry/61/statistic/6*](https://stat.gov.kz/official/industry/61/statistic/6) *and from the official statistical collection (http://www.rcrz.kz/index.php/ru/statistika-zdravookhraneniya-2). We used data for 2019-2020 (at the time of the study). The references are now listed in the reference list as 36-37.*

14. Line 108: Great that you’ve clarified that the schools are state owned/ran. Please ensure that you expand on this limitation in your discussion as private schools with children from higher socioeconomic backgrounds would likely have more myopia due to the link of socioeconomic status and myopia.

*We have included* *this in the discussion as follows: “The study was conducted in governmental schools only. Because a number of studies have shown the association of socioeconomic status with myopia [71–73], private schools with children from families with higher socioeconomic status would likely have more myopia.”.*

15. Line 116: you can obtain data on orthok patients refractive error, but I believe you mean refractive error without correction. Please rephrase.

*Thank you for your comment. The sentence revised and corrected to read: «Students under orthokeratology treatment were excluded because their uncorrected visual acuity and uncorrected refractive error could not be obtained.».*

16. Line 123: Why only ask about extracurricular near work, and how have you ensured that this has been answered accurately? Isn’t this a limitation?

*Thank you for your comment. This is a very important question, which we also studied when created the questionnaire. In the questionnaire, we listed the most common types of extracurricular near work (please see item 33 below). However, we agree that there may be certain limitations. We agree that this should be discussed as a limitation and have now added a sentence to the Limitations: “**With regard to classification of near work in the questionnaire we used, we only asked about extracurricular part of it, which may entail some classification bias and thus is another limitation of our presentation”.*

*Another limitation of our report with regard to the questionnaire we used is*

17. Line 121: Only in the discussion is the time of year for data collection mentioned. Many studies looking at time outdoors have had to adjust their questionnaires and methods etc. due to the seasonal effects of time outdoors. Please ensure you add what time of year the questionnaire was answered, as these will be needed to contextualise what the readers take from it.

*Thank you for highlighting this issue. We agree that the time of the year plays role, which is also related to the climate and different levels of insolation. The study was conducted between September to December. We have added this refinement to the methods and in the discussion section (paragraph 4, sentence 1 of Methods and sentence 4 in the Limitations paragraph).*

18. Line 127: were the people conducting the data collection the same clinical team throughout?

*Yes, the examination of all children in all schools and classes was carried out by one team (ophthalmologists and nurses). A sentence on that was added to the Examination protocol paragraph.*

19. Line 131: ‘specific background’. In what?

*This refers to the training of personnel for optometric examination of children and careful data recording. In accordance with the recommendations of RESC (protocol and manual of procedures to assessment of the prevalence of visual impairment attributable to refractive error or other causes in school children), a short training was held for the entire team at the study commencement. We've added a clarification to the Methods (the first paragraph of Examination protocol).*

20. Line 132: There is a great amount of detail about the lighting in the classroom. With cyclo this is not as relevant, and therefore I’m unsure if this level of detail is necessary, given that the detail in other aspects of the methods is not visible.

*Thanks for the comment. The study was conducted in classrooms, we included a description of the lighting for a general understanding of the conditions in which children learn. We have removed this paragraph as we agree this may be not of great value.*

21. Line 137: I believe they’re called Landolt C rings?

*Yes, they can be called Landolt ring or Landolt broken ring or Landolt C. We have edited the text accordingly.*

22. Line 141: pupil light reflex does not provide accommodation impact, would checking near vision have been better?

*We aimed to say that we measured the size of the pupil with a school ruler to verify complete dilation and cycloplegia. Refraction examination protocol in children recommends refraction assessment with a pupil wider than 6 mm. In addition, cycloplegia was verified by no pupil response to light.*

23. Line 145: I believe that the collection of data from the ‘worse eye’ is an extreme way to induce selection bias and potential over estimation of prevalence for refractive errors generally in the population. I would ask the editor and authors whether this process needs to change so that one eye was taken at random for the results using this data.

*In our opinion, choosing the best eye, focusing on the right or left eye, as well as random selection, seriously entails high risk of missing disorders that can lead to other disease, including impaired binocular vision, amblyopia, strabismus and disability. To better proceed with this discussion, we have now reviewed the literature and conclude that choosing the worst eye is a common practice, especially when assessing visual impairment in children. Furthermore, in accordance with the recommendations of the RESC, "Individuals were considered myopic if one or both eyes were myopic, and hyperopic if one or both eyes were hyperopic, so long as no eye was myopic.". Accordingly, for a child to be considered myopic or hyperopic, having refractive error unilaterally is sufficient. In other words, we aimed to identify pupils even if the pathology was in one eye only. This will allow better accuracy in estimating the total number of visually impaired children. However, we agree that if there is visual impairment in both eyes, choosing the worst eye will shift the SER and increase the number of severe cases. Hence, that was the rationale to include the worst eye in the analysis, even if this can entail some selection bias. In response to the pertinent comment of the Reviewer, we have elected to add one more sentence to the limitations as follows: “**Furthermore, some selection bias may be present because we looked at the worst eye to diagnose disorders”.*

24. Line 151: You’ve mentioned the IMI protocol papers for defining the limit of myopia, which is understandable. However, there are different thresholds for hyperopia, with no reasoning or references given. Please could this be clarified. The same goes for astigmatism.

*This is indeed a very important notation because the thresholds for astigmatism and hyperopia vary widely across studies. We reviewed the studies and found that hyperopia was defined as a  SER from + 0.5 to + 3.0D. Astigmatism in different studies was defined as cylindrical refraction from 0.5 to 1.5D. We chose to use the thresholds reported in the E3 - European Eye Epidemiology Consortium systematic review, which defined the threshold for hyperopia as SER ≥ 1.0D; and astigmatism threshold as cylindrical refraction ≥ 1.0 D in any meridian.*

<https://www.ncbi.nlm.nih.gov/pmc/articles/PMC4385146/>

*We have added a reference to the study in the Methods (Definitions).*

25. Line 164: Is the comment on the education department permission relevant, as I’m unsure if this is included in this study?

*The Department of Education in our country controls the work of schools. Permission from the city's Department of Education was required for the study to take place in schools, so we included it in the legal information section. Removed.*

26. Line 171: was the Mann Whitney test used even if the data was normally distributed?

*We are sorry that at some earlier stage of internal revision part of the text in statistical methods dropped out. Indeed, when data were normally distributed, we used t-test as an alternative for Mann-Whitney test. The sentence is now corrected to read: “Between-group comparisons of binary variables were tested using χ2 test from contingency tables, whereas continuous variables were tested using t-test or Mann-Whitney U-test depending on the distribution”.*

27. Line 175: I find it very surprising that there were no correlations between thing such as sports and time outdoors and near work and electronic use etc.

*This is indeed important, and we tried to consider multicollinearity in the models (outdoor time and sports activities; near work and full school day; near work and use of gadgets). Before we included the predictors in the adjusted model, we tested correlations using a correlation matrix. We could not use variance inflation factor or other tests of collinearity because of the binary nature of these variables. None of the correlations between variables included in the adjusted models exceeded 0.10; therefore, we failed to detect collinearity between predictors included.*

28. Results
    Table 1. The P values for male and female myopia are exactly the same, is this correct?

*That is an unfortunate typo. Thank you for your comment. Corrected.*

29. Line 200: are these differences between schools etc significant? Please provide a P value.

*Thank you for your comment. The differences were significant (p = 0.014), and p-value is now provided.*

30. Line 209: The levels and differences between them (or lack of) are not shown, as no P values have been done and detail is limited. This also goes for the comment on the prevalence being higher in 9th grade, you need P values.

*Thank you for your comment. We apologize for this omission. Changed the table and this paragraph, which now reads: “As can be seen from Table 3, there was an increase in the prevalence of all refractive errors and myopia in particular in older grades in both school types (p < 0.05). We did not observe an association between hyperopia and astigmatism with the grade of education”.*

31. Table 3: There is a * in the gymnasium part of 5th grade high myopia, but this isn’t replicated anywhere else, and so it implies nothing is significant. This doesn’t appear right with the numbers that are listed (greater numbers in year 9 for example), and it’s likely that significance is due to lower numbers.

*We are grateful for your important remark. In Table 3, we calculated the significance for the same grades but different types of schools. Indeed, significant differences were found only for high myopia among grade 5. However, we agree that this may be due to the lower number of children with high myopia. We added p-value to the table for clarity and a text description was added to the Results: When different types of schools were compared, there were no significant differences in the prevalence of refractive errors (myopia, hyperopia and astigmatism) between the corresponding grades of gymnasiums and secondary schools (p>0.05 for all grades).*

|  | Mean SER ± SD, D | Emmetropia, n (%) | Refractive errors, n (%) | Myopia, n (%) | High myopia, n (%) | Hyperopia, n (%) | Astigmatism, n (%) |
| --- | --- | --- | --- | --- | --- | --- | --- |
| General education schools | | | | | | | |
| 1^st^ | -0.11±1.11 | 316 (76.5) | 97 (23.5) | 72 (17.4) | 1 (1.4) | 25 (6.1) | 10 (2.4) |
| 5^th^ | -0.40±1.26 | 288 (71.6) | 114 (28.4) | 105 (26.1) | 1 (1.0) | 9 (2.2) | 9 (2.2) |
| 9^th^ | -0.84±1.70 | 242 (58.2) | 174 (41.8) | 162 (38.9) | 7 (4.3) | 12 (2.9) | 16 (3.8) |
| Gymnasium | | | | | | | |
| 1^st^ | -0.27±1.01 | 282 (79.2) | 74 (20.8)  p = 0.370 | 63 (17.7)  p = 0.924 | 1 (1.6)  p = 0.917 | 11 (3.1)  p = 0.053 | 7 (2.0)  p = 0.669 |
| 5^th^ | -0.57±1.60 | 253 (69.1) | 113 (30.9)  p = 0.446 | 102 (27.9)  p = 0.586 | 6 (5.9)*  p = 0.043 | 11 (3.0)  p = 0.506 | 13 (3.6)  p = 0.276 |
| 9^th^ | -1.08±1.99 | 187 (55.0) | 153 (45.0)  p = 0.382 | 144 (42.4)  p = 0.342 | 13 (9.0)  p = 0.069 | 9 (2.6)  p = 0.844 | 9 (2.6)  p = 0.360 |

*In the manuscript, we have replaced Table 3 with a comparison of the prevalence of refractive errors among different grades of education.*

32. Line 219: what about high myopia, that also increased?

*The risk of high myopia significantly increased in the 9th grade students (OR 10.42; 95% CI 2.43; 44.73). However, due to the limited number of cases of high myopia, 95% CI was so wide. We have added this point to the manuscript.*

33. Line 224: A supplementary material of the questionnaire, or full verbatim statements of how the questions were raised would be important. As your results for myopic parents don’t match with other studies and expectation, it’s likely down to data collection error. Therefore, this should be included in the methods, and stated as a limitation further in the discussion.

*Thank you, we agree that this information should have been added. The following information from the original questionnaire is now disclosed in the Methods:*

*-	“Does any parent have myopia?” with three response options (no; one of the parents; 'both parents). We did not specify whether that was a father or mother or even familial myopia.*

*-	“'How many hours does the child spend in the outdoor activities daily? “with three answer options (less than 1 hour a day, 1-2 hours a day, more than 2 hours a day).*

*-	“How many hours does the child spend on the near work (reading, drawing, handicraft, homework, etc.); time spent at school is not considered?” with three answer options (less than 1 hour a day, 1-2 hours a day, more than 2 hours).*

*-	“How many hours does the child spend with gadgets (computers, mobile phones, tabs, games, etc.), daily’, with four variants of answers (doesn’t use gadgets, less than 1 hour a day, 1-2 hours a day, more than 2 hours).*

*-	“Does your child attend sports club or section”, with two options to answer (yes, no)*

*-	“What school shift is your child in?” with two answer options (shift one, shift two or whole day.*

*In addition, we included gender and school grade. But due to the word limit in a manuscript, we decided to add the following sentence to the abstract: “The questionnaire included questions on the main risk factors such as parental myopia, screen time, time outdoors, sports activities, near-work, gender, grade and school shift”.*

34. Line 231: visual work is wrong, reword to near work. Also the tasks stated; these are not given earlier, how do the researchers know which tasks were included in the near work estimation? If these were measured directly or asked for, why have they not included this in more detail?

*Thank you for your comment. The sentence is revised and corrected to read: “Extracurricular near work over 2 hours per day increased the odds of myopia (OR 2.29; 95% CI 1.54; 3.42)”. In addition, we now describe the tasks of near work in the Methods, as stated above.*

35. Line 235: statements for comparisons would benefit from P values.

*Thank you for your comment. We added "p<0.01" to the sentence.*

36. Line 242: please reformat the equations and detail here.

*We have to admit that initially we were not intended to provide any formula to calculate the odds of visual impairment and make it a function of mathematics. We were advised to put this formula by one of the internal reviewers whom we showed the manuscript in search of advice. We personally consider citing this formula impertinent, and the Reviewer’s comment only strengthened this concern. Therefore, at this stage we have decided to completely delete the formula from the text as it looks misleading and overloads the reader with information he or she is not in need of.*

37. Table 4: the bands for low medium and high time outdoors and near work appear very rigid and with minimal distribution (2 hours and more than double the time 5 hours are in the same category for example). Where did the authors get their thresholds from?

*We agree with the Reviewer. However, when parents themselves fill the questionnaires in, they feel such clear time frames are more convenient for them to think of. When we made decision on what periods to use, we relied on data from previous studies, which also give hard time periods, including The North India Myopia Study (*[*https://www.ncbi.nlm.nih.gov/pmc/articles/PMC4342249/*](https://www.ncbi.nlm.nih.gov/pmc/articles/PMC4342249/)*); German KiGGS Study (*[*https://www.ncbi.nlm.nih.gov/pmc/articles/PMC8025934/*](https://www.ncbi.nlm.nih.gov/pmc/articles/PMC8025934/)*); Study by Stepan Rusnak et al. (https://www.ncbi.nlm.nih.gov/pmc/articles/PMC5859838/); Xiaoyan Wu et al. (*<https://www.ncbi.nlm.nih.gov/pmc/articles/PMC5006500/>).

38. Line 258: It is as expected, but likely down to age, this should be discussed too.

*Thank you for your comment. We have expanded the discussion of myopia with age in the Discussion section. This insertion reads: “**Furthermore, our findings were consistent with other studies in children confirming growing prevalence of myopic refraction and a decrease in the rate of hyperopia with age, especially in the second decade of life [8,47–55]”.*

39. Line 278: the Local studies are discussed. Could the refractive thresholds used in these studies be mentioned?

*Thanks for the clarification. Differences in thresholds were mainly for hypermetropia, which was defined as more then SER + 1.25 D. In addition, there were variations in methodology (retinoscopy vs autorefraction) and the definition of refractive errors only by the spherical component. We have added these points to the Discussion: “For example, refractive errors in previous studies were only determined by a spherical refraction component and equivalent refraction, but not SER. In addition, refraction was measured using retinoscopy as opposed to autorefraction [31–35]. Although both methods are reliable [45,46], direct comparison between studies may not be easy”.*

40. Line 281: statement on threholds etc. throughout this paragraph need evidencing with references.

*Thank you for your comment. Done. Please see item above citing the text inserted.*

41. Line 284: Big claim that the study is important to develop a prevention plan. Issues with similar statements have been given before. All that the study is able to say is that myopia prevalence is higher than other refractive errors, and that this may be cause for concern. Not for developing a prevention plan.

*Thank you. This phrase has been removed.*

42. Line 300: Please evidence where sport itself when not a substitute for time outdoors has been shown to protect against myopia. This is generally considered a tenuous link.

*We thank the Reviewer for catching this up and addressing this important aspect. We have to admit that we did not distinguish between outdoor activities and sports, and only asked about outdoor activities. Hence, referring to sports is inappropriate and should be avoided. We have decided to delete the term ‘sport’ and retain ‘time spent outdoors’.*

43. Line 305: limitation of questionnaires discussed, quotes and exerts as mentioned above are needed.

*Thank you. We have added a paragraph about the limitations of our study*

44. Line 313: What is RESK? Definition needed

*RESC definition is provided in the first sentence of the third paragraph in Methods as follows: “This cross-sectional school-based study was performed according to the protocol of Refractive Error Study in Children (RESC). This protocol was designed to standardize the methodology used to obtain prevalence data on childhood refractive errors”.*

45. Line 318: Here it now only apparent that 50% of participants responded for the questionnaire. This is not apparent at all from the study and methods and results, and therefore can be misleading. This needs to be clearer, and likely means that you only have a subset population that you’ve done analysis for risk factors on, not the full sample.

*Thanks for the comment. The low response rate is also a limitation of this study. We apologize for not informing the reader about 50% response rate earlier in the manuscript. And we have now decided to put this data in the Results: “**Response rate to the questionnaires filled out by the parents on behavioral risk factors and the role of parental myopia was 50.9% (1166 subjects)”.*

46. Line 328: Conclusion phrasing is atypical e.g. starting with thus, and claims of the serious health concern are bold and unfounded. This study did NOT demonstrate that it is a serious health problem, it just demonstrates prevalence and linked potential risk factors. Comments on why it causes health concerns or worries for healthcare are not discussed at all, and no protective factors are able to be measured. The whole section needs rewriting and a more conservative approach for what the paper has done and why it is useful.

*Thank you for your comment. We agree with this remark and amended the paragraph to make out conclusions not sound too strong and present only those directly arising from the study findings. Now this paragraph reads: “In summary, our study demonstrated that more than a third of school-age children in Almaty had refractive errors, whereas myopia was most common. Compared to previous local studies in this age group, myopia prevalence among urban children likely increased over the past 10 years. We also found that time spent outdoors may be associated with reduced odds of myopia; however, the effects we identified should be further studied in rural population of Kazakhstan.”.*

We are grateful to the editor and reviewers for their time and all the valuable comments, as well as for the help in improving our manuscript. We will make every effort to meet the requirements of the Journal.

Corresponding author, Denis Vinnikov
