# Supplementary material for: Prevalence of refractive errors and risk factors for myopia among schoolchildren of Almaty, Kazakhstan: A cross-sectional study
Source: PLoS One. 2022 Jun 3;17(6):e0269474. doi: 10.1371/journal.pone.0269474 (PMC9165805; doi:10.1371/journal.pone.0269474)
Supplement: Attachment — Submitted filename: Point-by-point response to reviewers.docx [file pone.0269474.s002.docx]

PONE-D-21-32174R1

Prevalence of refractive errors and risk factors for myopia in schoolchildren of Almaty, Kazakhstan: a cross-sectional study

PLOS ONE

To the Editor:

We would like to thank the reviewers and the Editor for comments and help in improving our manuscript. In the following detailed response, we address each critique calling for changes point-by-point, indicating where relevant additional text has been added to the body of the manuscript and its location. We hope that this has improved the manuscript.

ACADEMIC EDITOR: Please pay particular attention to the point regarding eye selection for statistical analysis. If sufficient evidence to support the approach used cannot be provided, then please consider reanalysis of data using approaches more aligned with established norms.

*Thank you for your comment. In accordance with the recommendations of the WHO and RESC, "Individuals were considered myopic if* ***one*** *or both eyes were myopic, and hyperopic if one or both eyes were hyperopic, so long as no eye was myopic.". Accordingly, for a child to be considered myopic or hyperopic, having refractive error unilaterally is sufficient. In our opinion, choosing the best eye, focusing on the right or left eye, as well as random selection, seriously entails high risk of missing disorders that can lead to other disease, including impaired binocular vision, amblyopia, strabismus and disability. To better proceed with this discussion, we have now reviewed the literature and conclude that choosing the worst eye is a common practice, especially when assessing visual impairment in children. For example, in 2006, the population multiethnic study of pediatric eye diseases (MEPEDS) was introduced, in which the determination of visual acuity and refraction was carried out in the worst eye (*[*https://pubmed.ncbi.nlm.nih.gov/16877284/*](https://pubmed.ncbi.nlm.nih.gov/16877284/) *).*

*In many large studies, the selection of the worst eye is stated explicitly. In other studies, in order to be considered as a subject with refractive error, the participant had to have a refractive error in at least one eye. In cases with one myopic and a fellow hyperopic eye, the refractive error of the eye with larger absolute SE was taken into account. Which also means choosing the worst eye. These are just some of them:*

[*https://www.ncbi.nlm.nih.gov/pmc/articles/PMC6941318/*](https://www.ncbi.nlm.nih.gov/pmc/articles/PMC6941318/)

[*https://www.ncbi.nlm.nih.gov/pmc/articles/PMC6029024/*](https://www.ncbi.nlm.nih.gov/pmc/articles/PMC6029024/)

[*https://pubmed.ncbi.nlm.nih.gov/25626973/*](https://pubmed.ncbi.nlm.nih.gov/25626973/)

[*https://www.ncbi.nlm.nih.gov/pmc/articles/PMC5468969/*](https://www.ncbi.nlm.nih.gov/pmc/articles/PMC5468969/)

[*https://www.ncbi.nlm.nih.gov/pmc/articles/PMC8211073/*](https://www.ncbi.nlm.nih.gov/pmc/articles/PMC8211073/)

[*https://www.ncbi.nlm.nih.gov/pmc/articles/PMC8139091/*](https://www.ncbi.nlm.nih.gov/pmc/articles/PMC8139091/)

[*https://pubmed.ncbi.nlm.nih.gov/12395920/*](https://pubmed.ncbi.nlm.nih.gov/12395920/)

[*https://www.ncbi.nlm.nih.gov/pmc/articles/PMC7197133/*](https://www.ncbi.nlm.nih.gov/pmc/articles/PMC7197133/)

[*https://www.ncbi.nlm.nih.gov/pmc/articles/PMC2815146/*](https://www.ncbi.nlm.nih.gov/pmc/articles/PMC2815146/)

[*https://www.ncbi.nlm.nih.gov/pmc/articles/PMC5468969/#CR22*](https://www.ncbi.nlm.nih.gov/pmc/articles/PMC5468969/#CR22)

[*https://www.ncbi.nlm.nih.gov/pmc/articles/PMC3853776/*](https://www.ncbi.nlm.nih.gov/pmc/articles/PMC3853776/)

[*https://journals.plos.org/plosone/article?id=10.1371/journal.pone.0120764*](https://journals.plos.org/plosone/article?id=10.1371/journal.pone.0120764)

[*https://www.ncbi.nlm.nih.gov/pmc/articles/PMC8453643/*](https://www.ncbi.nlm.nih.gov/pmc/articles/PMC8453643/)

*Moreover, in none of the above studies, the choice of the worst eye was a limitation.*

*All of the above explains the rationale for choosing the worst eye for examination. However, we agree that if there is visual impairment in both eyes, choosing the worst eye will shift the SER and increase the number of severe cases. In response to the pertinent comment of the Reviewer, we added the following paragraph to the Materials and Methods section: "**Because the correlation of spherical equivalent and visual acuity between the eyes was high (Pearson correlation coefficient 0.91 (95% CI 0.90; 0.92; p<0.001)), the spherical equivalent from the worse eye was used for analysis, similar to several previous cross-section studies in children."*

Reviewer #2:

1. Comment number 16 talking about limitations to the questionnaire, it appears that your response was truncated, when commenting on another limitation. Please could the authors state whether there is something else to comment on here, as these words cannot be seen after the new section written in the manuscript.

*Thank you for your comment. We acknowledge that we made a misprint in point 16. The phrase “Another limitation of our report with regard to the questionnaire we used is…” is the beginning of an answer to another question that erroneously fell into this paragraph. We apologize for the annoying misprint. The answer should have been: «Thank you for your comment. This is a very important question, which we also studied when created the questionnaire. In the questionnaire, we listed the most common types of extracurricular near work (please see item 33 below). However, we agree that there may be certain limitations. We agree that this should be discussed as a limitation and have now added a sentence to the Limitations: “With regard to classification of near work in the questionnaire we used, we only asked about extracurricular part of it, which may entail some classification bias and thus is another limitation of our presentation”.*

We have made these changes to the manuscript (line 317)

2. I believe that having understood the questions asked within the questionnaire, that there is a new limitation to add, unfortunately. Many children will not know what myopia is (indeed, most of the adult population may not either), and may usually know some form of non-scientific term such as ‘short-sightedness’ or similar. Given this wording to identify myopic parents, it may be that children did not understand the question, and this may be why the result on number of myopic parents may be not as expected. This should therefore be added to the discussion in this relevant part, as a limitation and a reason for the results found.

*Thank you for your comment. We completely agree with you on this remark. We wrote about this in the discussion section: "Besides, there might have been a lack of parental awareness; some respondents could have mistaken for myopia any refractive errors requiring spectacle correction, such as presbyopia or hyperopia". We now decided to add a clarification on the terminology as follows: "**In addition, it was likely that not all respondents knew the term 'myopia', instead using non-scientific 'nearsightedness' or 'short-sightedness', which could also affect the results of the study"*

3. I appreciate the authors answer to point 22 on cycloplegia. No response to light is indeed one of the signs for potential cycloplegia. However, usually protocols for cyclo-autorefraction indicate a wait time also after the instillation of drops (sometimes more than one), for the reason that just stopping pupillary reactions is not enough to demonstrate there is no accommodation. Could the authors clarify if there was a wait time, or just if pupil reaction and size was only checked.

*Thank you for the clarification, we probably misunderstood the question. We agree that exposure time is important for cycloplegia. In our study, the wait time after the instillation of drops was 30 minutes. We have described this in the 'Materials and Methods' section, paragraph 'Examination protocol': "We measured cycloplegic refraction 30 minutes after the instillation of a drop of cyclopentolate 1% twice."*

4. For the response to point 23, I appreciate the response from the authors to answer the fact that they have used the worse eye, and added a point to the limitations, however for myopia-related studies, this is not typical, and may be seen as strange. Many researchers would firmly state that this would inflate all values found and ask why the authors didn’t do an analysis of just one eye, and believe that refractions/eye condition prevalence would level out due to laterality chance. Indeed, the authors have said they’ve reviewed literature that showed this was best approach. To placate the anticipated readership, please could they add this to their methods as explanation for their approach, along with references they’ve found to back this approach. In conclusion, I understand where the authors are coming from, yet I still have reservations. I leave this up to the editor to decide what to do.

*We are grateful to you for your attention to this issue. We agree that this is a significant limitation of our study, and we must explain the choice of the worst eye for analysis. Please see clarification and rationale provided in response to the Editor’s comment above. To do this, we added the following paragraph to the Materials and Methods section: "Because the correlation of spherical equivalent and visual acuity between the eyes was high (Pearson correlation coefficient 0.91 (95% CI 0.90; 0.92; p<0.001)), the spherical equivalent from the worse eye was used for analysis, similar to several previous cross-section studies in children"*

5. Point 27, thank you for clarifying. However, are you sure that the variables are binary? Do you mean categorical? And if so, did you only categorise in a yes/no binary format? Apologies if I have misunderstood this.

*We are sorry for using improper term. Yes, we meant categorical variables and we have now corrected ‘binary’ to ‘categorical’. Some of these were categorized into ‘yes/no’, but other into categories, depending on the variable.*

6. Point 28. Thanks for clarifying. Unfortunately the new manuscript demonstrates you’ve removed the female p value, but I cannot see the correct one.

*We are truly sorry for choosing improper format for data presentation in Table 1. We have now edited the Table to make it self-explanatory.*

*We have also noticed that the format should also be amended in Table 2 and have done that to be consistent with Table 1.*

We are grateful to the editor and reviewers for all further valuable comments, as well as for the help in improving our manuscript. We will make every effort to meet the requirements of the Journal.

Corresponding author, Denis Vinnikov
